# Analysing nystagmus waveforms: a computational framework

**DOI:** 10.1038/s41598-021-89094-7

**Published:** 2021-05-07

**Authors:** Richard V. Abadi, Ozgur E. Akman, Gemma E. Arblaster, Richard A. Clement

**Affiliations:** 1 Faculty of Biology, Medicine and Health, University of Manchester, Manchester, M13 9PT UK; 2 College of Engineering, Mathematics and Physical Sciences, University of Exeter, Exeter, UK; 3 Orthoptics Department, NHS Foundation Trust, Sheffield Teaching Hospitals, Sheffield, UK; 4 Division of Ophthalmology and Orthoptics, Health Sciences School, University of Sheffield, Sheffield, UK

**Keywords:** Computational biology and bioinformatics, Neuroscience, Systems biology, Medical research

## Abstract

We present a new computational approach to analyse nystagmus waveforms. Our framework is designed to fully characterise the state of the nystagmus, aid clinical diagnosis and to quantify the dynamical changes in the oscillations over time. Both linear and nonlinear analyses of time series were used to determine the regularity and complexity of a specific homogenous phenotype of nystagmus. Two-dimensional binocular eye movement recordings were carried out on 5 adult subjects who exhibited a unilateral, uniplanar, vertical nystagmus secondary to a monocular late-onset severe visual loss in the oscillating eye (the Heimann-Bielschowsky Phenomenon). The non-affected eye held a central gaze in both horizontal and vertical planes (± 10 min. of arc). All affected eyes exhibited vertical oscillations, with mean amplitudes and frequencies ranging from 2.0°–4.0° to 0.25–1.5 Hz, respectively. Unstable periodic orbit analysis revealed only 1 subject exhibited a periodic oscillation. The remaining subjects were found to display quasiperiodic (*n* = 1) and nonperiodic (*n* = 3) oscillations. Phase space reconstruction allowed attractor identification and the computation of a time series complexity measure—the permutation entropy. The entropy measure was found to be able to distinguish between a periodic oscillation associated with a limit cycle attractor, a quasiperiodic oscillation associated with a torus attractor and nonperiodic oscillations associated with higher-dimensional attractors. Importantly, the permutation entropy was able to rank the oscillations, thereby providing an objective index of nystagmus complexity (range 0.15–0.21) that could not be obtained via unstable periodic orbit analysis or attractor identification alone. These results suggest that our framework provides a comprehensive methodology for characterising nystagmus, aiding differential diagnosis and also permitting investigation of the waveforms over time, thereby facilitating the quantification of future therapeutic managements. In addition, permutation entropy could provide an additional tool for future oculomotor modelling.

## Introduction

Visual perception is strongly dependent on the stability of the two eyes^1^. An unsteady involuntary ocular oscillation is called a nystagmus. A nystagmus may be present at birth or develop within the first months of life (congenital/infantile: early-onset)^2–4^ or be acquired (late-onset)^4^. A diverse number of nystagmus waveform types have been described in the clinical literature, with studies exploring how the nystagmus intensity (amplitude × frequency)^5–7^, nystagmus waveform^8, 9^ and the foveation dwell time^5, 10–14^ can influence visual acuity. Standard clinical assessments of nystagmus involve recording the laterality, conjugacy, plane of oscillation, amplitude, frequency, waveform shape and the foveation dwell time.

Outside the traditional clinic setting, additional techniques have been used. A linear systems analysis has been used to determine the periodic nature of the waveforms by means of both Fourier^8, 15–17^ and wavelet analysis^18, 19^. Results of a principal components analysis of early-onset nystagmus supported the hypothesis that nystagmus waveforms form a continuum, rather than falling into discrete waveform classes^20^.

More recently, nonlinear dynamical systems theory has provided additional important computational advances^20–31^. The basis of this dynamical approach is to geometrically represent the states of a nystagmus time series by computing the trajectories (or attractors) in phase space^32, 33^. A stable system is then represented by a stable fixed point (a point attractor) whilst a range of other attractors define the different nystagmus waveform types. Significant developments have included the mapping of nystagmus waveform attractors^20, 22, 23, 25–30^, descriptions of the transitions from one attractor type to another (bifurcations)^26, 27, 29, 34^, the quantification of foveation^28, 35^ and the measurement of the dimensionality underlying the waveforms^22, 25, 27^. The application of nonlinear analysis has also aided the modelling of the underlying neural behaviour responsible for the oscillations^22, 23, 26–29, 36^.

With these background studies in mind, we believe it is now timely to present a new computational framework for analysing nystagmus oscillations. Specifically, we intend to incorporate within the framework a quantitative measure of the complexity of the nystagmus waveform—the permutation entropy index^37–39^.

**Figure 1:**
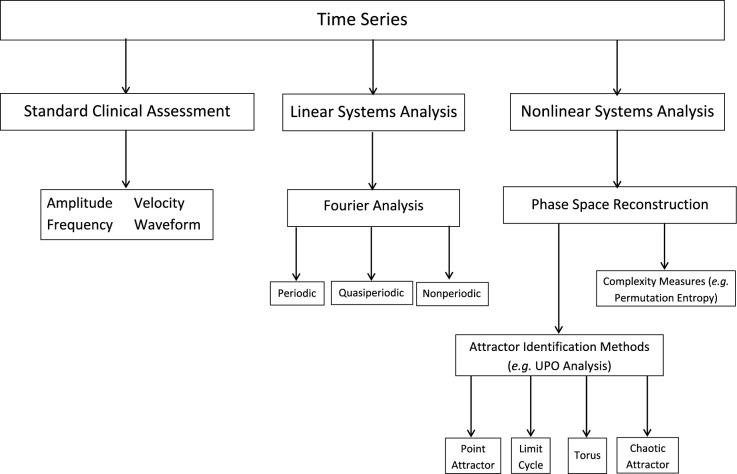
A computational framework for analysing a nystagmus time series that combines linear and nonlinear investigative methods to supplement the standard clinical assessment. The linear and nonlinear systems analyses are based on spectral decomposition and attractor reconstruction respectively.

Our analytical framework is presented in Fig. 1. It has 3 components: a standard clinical assessment, a linear systems component (Fourier analysis) and a nonlinear systems component (unstable periodic orbit and permutation entropy analyses). The deterministic behaviour of the nystagmus is characterised by the unstable periodic orbits (UPOs) of the oscillations^24^, whilst the permutation entropy provides a measure of the complexity of the waveforms^37^. In the past, the clinical assessment of the waveform complexity has been subjectively approximated by viewing the shape of the nystagmus waveform and/or noting the regularity of the low-velocity foveation periods within the waveform traces. Here we propose that a permutation entropy analysis will provide a substantial additional numerical scaling of the waveform complexity, thereby providing clinicians with a new means to not only assist in the selection of patients for therapy, but also indicate whether the therapy has been successful.

## Methods

### The study cohort: inclusion and exclusion criteria

We chose to study a specific late onset acquired oculomotor gaze disorder secondary to the loss of vision in one eye only—the Heimann-Bielschowsky phenomenon^40–43^. The pertinent and consistent clinical features of individuals presenting with a HBP are a uniocular, uniplanar, vertical nystagmus in the eye with visual loss. We selected this particular nystagmus syndrome since it is exclusively associated with a static late-onset severe visual loss in subjects without any history of a previous nystagmus. Most importantly, there are no detectable pathologies, apart from the single event that caused the initial and sustained visual loss. In this way, our cohort belongs to a discrete homogeneous category. In addition, we intentionally set ourselves the challenge of using our framework to investigate low frequency vertical oscillations, which often introduce difficulties with the recording and analysis of the nystagmus.

All subjects had a sustained late-onset monocular visual loss that was classed as severe or worse (visual acuity ≤ 20/200^44^) and which was solely confined to the affected eye. Exclusion criteria included accompanying neurological disorders, multiple causes of the visual loss, conditions often associated with an early-onset visual loss (*e.g*. strabismic amblyopia, spasmus nutans, infantile nystagmus and infantile cataract), significant head postures, and the presence of non-physiological ocular oscillations prior to the onset of the visual loss.

Informed consent was obtained from all subjects after the nature of the study had been explained. Ethical approval was granted by the Ethics Committee of the University of Manchester. Experimental and clinical protocols adhered to the tenets of the Declaration of Helsinki.

### Subjects and the clinical evaluation

Five adults (1 male and 4 female: age range 33–46 years) took part in the study. A comprehensive visual examination was carried out, including a full general, ocular and neurological history. Refractive state, visual acuity, binocular status, ocular motility and ocular alignment were measured. The magnitude (spatial resolution), the onset time and the duration of the visual losses were recorded.

### Eye movement recording

Horizontal and vertical eye movements were monitored using 2 separate recording systems for each of the 5 subjects whilst they binoculary viewed the targets.

1. A head mounted infrared IRIS 6500 limbal tracker system (Skalar Medical, Delft, The 1 Netherlands). The analogue output was filtered through a 100 Hz low pass filter, digitised to 12-bit resolution and then sampled at intervals of 5-ms (200 Hz). The system was linear over a range of ± 20°, with a resolution of 0.1°.
2. A 3-dimensional head-mounted video-based infra-red pupil tracking eye tracker running at 400 Hz with a resolution of 0.1° or better. (Chronos: Skalar Medical, Delft, The Netherlands).

Both recording methods were chosen to avoid the methodological limitations associated with more invasive techniques (such as the search coil) which would invariably rule out protracted and multiple recording sessions with our subjects, some of whom had also experienced anterior eye trauma.

Subjects sat in a dimly lit room equivalent to a mesopic level of illumination. Eye movements were calibrated by using either (1) a projected 2° circular stimulus that moved at 0.3 Hz horizontally or vertically over a range of ± 10°, and/or (2) a 10° × 10° stationary projected grid. Subjects were instructed to either follow the stimulus motion or to saccade between specific locations on the stationary grid as accurately as possible.

Fixation stability in primary gaze was assessed during attempted binocular fixation of a stationary 5.5° bull’s eye target, which had a large cross passing through its centre. Ocular alignment was assessed using the cover test with the bull’s eye target and/or a laser light source target. Throughout all recording sessions, a chin rest and supplementary cheek supports were used to stabilise the head. Eye movement data was stored and analysed off-line using custom-written MATLAB software. Fixation recording runs lasted between 40 and 60 s and were repeated on at least two occasions during each visit.

Separate from the laboratory-based fixation and eye alignment studies, eye movements were also externally filmed (JVC DV-j70 camcorder) to gather additional real time information.

### Data analysis

#### The standard clinical assessment

The means and standard deviations of the horizontal and vertical eye position traces were computed for both the visually affected eye and its fellow. In addition, chart recordings were subjectively examined to determine the nystagmus intensity, baseline drift and waveform regularity throughout individual recording sessions. Here, the amplitude of a nystagmus is defined as the magnitude of the change in eye position with each oscillation. Frequency is defined as the time between the peak-to-peak excursions of the oscillation. Our initial analysis was to subjectively establish whether the oscillations appeared periodic or nonperiodic. This differentiation proved very challenging, particularly as the oscillation frequency often fell below 0.5 Hz. Here, we define a periodic oscillation as a change of eye position that is cyclic, with a fixed period, whereas a nonperiodic oscillation is acyclic (*e.g*. quasiperiodic or nonperiodic).

#### The Fourier analysis

Fourier amplitude spectra were computed on successive segments of the eye movement recording as previously described^24^. We used a window length of 4000 data points, corresponding to a recording length of 20 s, which were weighted by a triangular function of position for waveform smoothing, and padded at either end by 1000 zero values to minimize aliasing. These operations provided a frequency resolution of 0.033 Hz. It should be noted that since the oscillations exhibited were of a low frequency, we adopted a longer window length than we had used in previous studies with early-onset/infantile nystagmus, where the oscillations were horizontal and of a far higher intensity.

#### The unstable periodic orbit (UPO) analysis

Unstable periodic orbits (UPOs) were identified in the data by a fixed-point technique introduced and developed by So and his colleagues^45, 46^. In this technique, the period of each cycle of an oscillation is obtained from the interval between threshold crossings (*i.e.* the times at which the waveform passes through a fixed (threshold) value). A linear model of successive interval lengths was used to transform the lengths into estimates of the period of the underlying orbit. The period was identified by a peak in the histogram of the transformed data and its significance was tested by comparison with surrogate data obtained by shuffling the interval data. The widths of the histogram bins were set at 0.05 s. Representative examples of periodic behaviour were found by establishing which sequences of intervals approached the identified period most closely, before subsequently deviating from it^24, 35^.

#### Attractor reconstruction

The behaviour of a dynamical system can be described by a set of differential equations governing the temporal dynamics of the system^32, 33, 47^. The dynamical evolution of the system corresponds to a series of consecutive points in phase (or state) space, referred to as the trajectory of the system, and the corresponding graphical depiction is referred to as the phase portrait. The region of state space to which the trajectories of a dynamical system converge is known as an attractor. Transitions from one attractor to another occur when the control parameter of the system passes through a critical value. This qualitative change is called a bifurcation^33, 48^.

**Figure 2:**
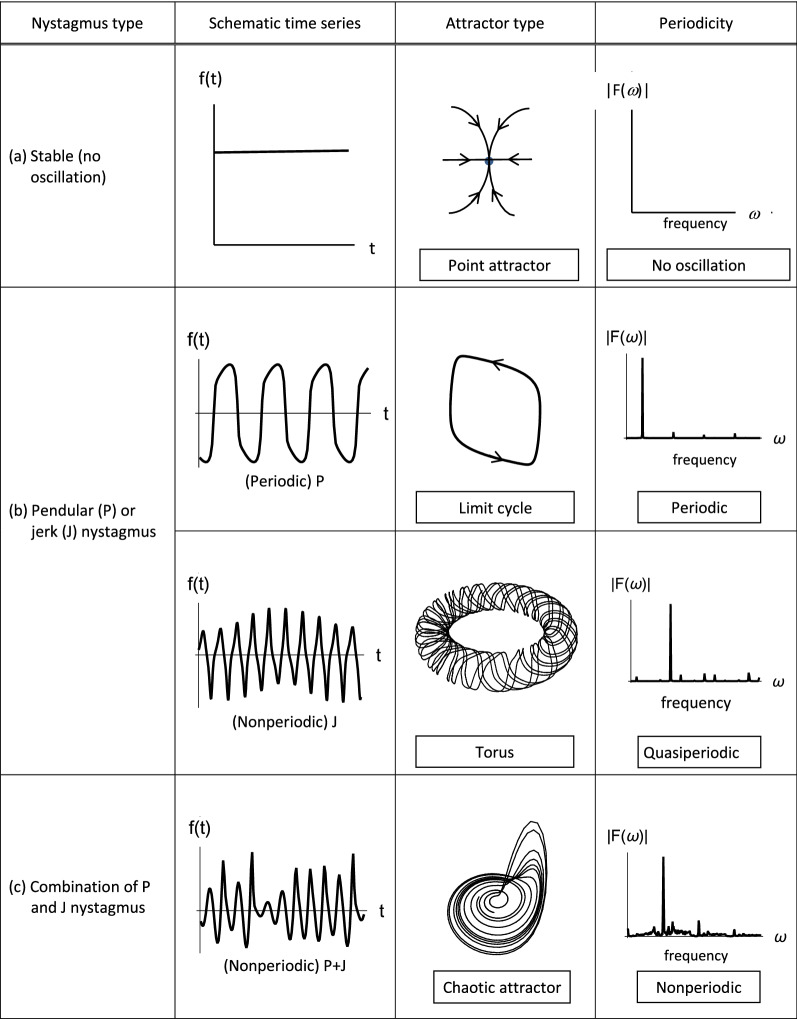
A comparison between stable (first row (**a**)) and unstable fixation (second row (**b**) and third row (**c**)). Columns 2, 3 and 4 show the corresponding time series, attractor type and frequency spectra, respectively. Note that all phase trajectories converge to the attractor over time: (**a**) A system in a state of stable equilibrium (*e.g.* steady fixation) is represented by a fixed point. Unstable systems (giving rise to unsteady fixation) are illustrated by ((**b**)-top) a one-dimensional limit cycle (periodic oscillation), ((**b**)-bottom) a two-dimensional torus (quasiperiodic oscillation) and (**c**) a higher-dimensional chaotic attractor (nonperiodic oscillation).

The simplest stable solution of a dynamical system is a stable fixed point, and with increasing time, all trajectories terminate at this point (Fig. 2a). Stable fixed points are thus static point attractors and describe the normal behaviour of the oculomotor control system during steady gaze^22, 27^. The system is accordingly deemed to be in a single permanent state and in equilibrium. On the other hand, unstable dynamical systems yield trajectories in state space that do not converge to a point attractor. Possible attractors include one-dimensional closed loops (a limit cycle) (Fig. 2b-top), two-dimensional doughnut-shaped surfaces (a torus) (Fig. 2b-bottom) and, three or more dimensional topologies (*e.g.* strange or chaotic attractors) (Fig. 2c)^22, 25, 27, 34, 49^.

The UPOs identified in “The unstable periodic orbit (UPO) analysis” section were used to reconstruct the corresponding attractors from each subject’s waveform^29^. Attractor reconstruction was implemented using the delay embedding procedure, in which a window of length *d* (referred to as the embedding dimension) is slid across the time series {*x*(*i*), *i* = 1,2,…}, yielding delay vectors {***X***_*i*_ = [*x*(*i*), *x*(*i* + 1),…, *x*(*i* + (*d*−1))], *i* = 1,2,…}. The delay vectors reconstruct the trajectory of the underlying dynamical system from which the time series was measured in a *d*-dimensional space (provided that *d* is sufficiently large), and thus provide a means of classifying the corresponding attractor (*e.g.* as a limit cycle, torus etc*.*—see Fig. 2)^30, 50–52^. Previous studies have shown that an embedding dimension *d* of 7 is sufficient to capture the variance in oculomotor tremor time series^22, 26, 29^. Accordingly, the same embedding dimension was applied to the segments of each subject’s time series that most closely followed the UPO. The delay vectors generated in this way were then projected onto their first three principal components in order to visualise the reconstructed attractor^22, 26, 29, 52^.

#### Permutation entropy analysis

Permutation entropy analysis determines the level of predictability within a time series to yield a scalar measure of waveform complexity^37–39^. Permutation entropy is simple and fast to compute and is also fairly robust to dynamical and observational noise. As such, it is well-suited to the analysis of physiological time series^39^, and the low intensity, uniocular, vertical oscillations displayed by our subjects. This technique yields similar information to the Lyaponov exponent on the stability of a dynamical system, whilst being more readily applicable to real-world data^37^.

The permutation entropy is calculated from the delay vectors {***X***_*i*_ = [*x*(*i*), *x*(*i* + 1), …, *x*(*i* + (*d*−1))], *i* = 1,2,…} generated from the time series {*x*(*i*), *i* = 1,2,…}with an embedding dimension *d* as follows^37, 38^. Each delay vector ***X***_*i*_ is uniquely mapped to the symbol sequence of length *d*, [*j*_1_, *j*_2_, …, *j*_*d*_], that encodes its arrangement into increasing order, *i.e.* such that: *x*(*i* + *j*_1_−1) ≤ *x*(*i* + *j*_2_−1) ≤ … ≤ *x*(*i* + *j*_*d*_−1) (for example, in a 3-dimensional delay space, the delay vector [0.1, 3, 10.5] would be mapped to the symbol sequence [1, 2, 3] and the delay vector [1.2, −4, 3] would be mapped to the sequence [2, 1, 3]). Then if *K* ≤ *d*! is the number of distinct symbol sequences obtained from the delay vectors and the probabilities of these symbol sequences are *P*_1_, *P*_2_, …, *P*_*K*_, the permutation entropy of the time series is defined as.

$$H_{p} = - \mathop \sum \limits_{j = 1}^{K} P_{j} \ln P_{j} .$$

**Figure 3:**
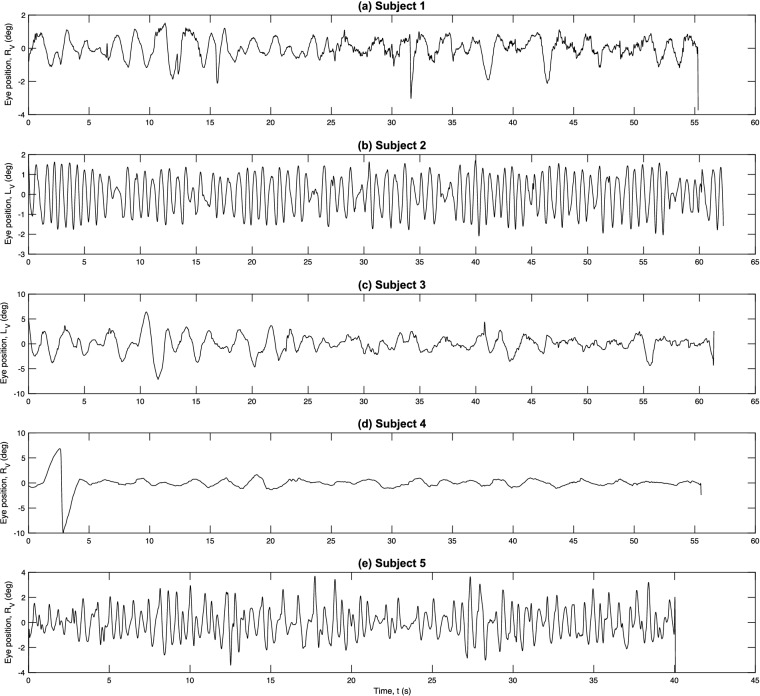
Time series used for the permutation entropy calculations (see text for further details).

*H*_*p*_ attains its minimum value of 0 when there is only one symbol sequence (*K* = 1 with *P*_1_ = 1) and its maximum value of ln(*d*!) when all sequences occur with equal probability (*K* = *d*! with *P*_*j*_ = 1/*d*!) Thus, it is standard to normalise *H*_*p*_ by ln(*d*!), giving a measure *h*_*p*_ = *H*_*p*_/ln(*d*!) for which 0 ≤ *h*_*p*_ ≤ 1. The quantity *h*_*p*_ then gives a measure of the complexity of the time series, with a larger value of *h*_*p*_ indicating a more irregular time series^37, 38^. Here, prior to the calculation of *h*_*p*_, the moving average of the signal was subtracted to eliminate baseline drift^51^. The resulting waveforms are shown in Fig. 3. Following the approach used for EEG analysis in^38^, for each time series the permutation entropy *h*_*p*_ was then calculated for all overlapping windows of a fixed length, *T*_w_. The value of the embedding dimension *d* was set to 7 (the same value used for attractor reconstruction) and *T*_w_ was set to 25.2 s to ensure that all symbol sequences possible with this *d* value could potentially be observed in a single window.

## Results

### The standard clinical assessment

A summary of the clinical details of the subjects are shown in Tables 1 and 2. All subjects presented with a late onset acquired uniocular, uniplanar (vertical) nystagmus that substantially reduced form perception. LogMAR visual acuity losses fell into one of three categories: severe (≤ 20/200; subject 2), profound (≤ 20/1000; subject1) and no light perception (NLP) (subjects 3, 4 and 5). The age at which vision loss occurred ranged between 6 to 46 years, with the lowest onset age beyond the critical period for form perception^53^. The duration of the visual loss prior to the onset of the nystagmus ranged between 1–24 years, with a median value of 2 years. Ocular alignment was compromised in 4 of the 5 subjects. The visually non-affected eye of each of the 5 subjects held a central gaze within ± 10 min of arc in both horizontal and vertical planes. Fixation was therefore deemed stable and not significantly different from normal.

**Table 1:** Summary of the clinical findings from the 5 subjects.

Patient		Sensory status				Ocular motor status				
Subject	Age:Sex	Affected eye: VA	Age at time of visual loss	Duration of monocular visual loss prior to onset of fixation instability	Cause of monocular visual loss	Ocular alignment	Age at time of fixation instability	Binocular fixation behaviour		
								R	L	Mean amplitude and frequency of affected eye
1	34Y:M	R:20/1000	26	2	Ocular trauma	Residual XT	28	↕	●	4.0° 0.5 Hz
2	33Y:F	L:20/200	6	2	Ocular trauma	Residual XT	8	●	↕	2.6° 1.5 Hz
3	54Y:F	L:NLP	46	1	Optic nerve tumour	No deviation	47	●	↕	2.5° 0.5 Hz
4	46Y:F	R:NLP	18	24	Venous thrombosis	Residual ET	42	↕	●	2.0° 0.25 Hz
5	41Y:F	R:NLP	26	6	Uveitis	Secondary XT	32	↕	●	3.5° 1.2 Hz

VA = visual acuity. M = male. F = female. R = right eye. L = left eye. NLP = no light perception. Age = age at the time of the final eye movement recording session in years. Residual tropia = a smaller deviation (in the same direction) following extra-ocular muscle surgery; secondary tropia = a strabismus secondary to a visual loss. ET = esotropia. XT = exotropia. ° = degrees. Hz = Hertz. ● = stable fixation. ↕ = vertical oscillation. Note: The fixation pictograms indicate fixation behaviour. Frequencies and amplitudes in the rightmost column were approximated directly from chart recordings by experienced oculomotor clinicians. All 5 subjects exhibited their oscillations for a minimum period of 4 years prior to the commencement of the study.

**Table 2:** Summary of the linear and nonlinear analyses of the nystagmus waveforms from each subject.

Subject	Oscillation frequency (Hz)			Periodicity category			Permutation entropy
	Clinical assessment	Fourier analysis	UPO analysis	Periodic	Quasi-periodic	Non-periodic	
1	0.50	0.49	0.67			✓	0.2571 (0.0184)
2	1.50	1.41	1.30	✓			0.1477 (0.0014)
3	0.50	0.40	0.47			✓	0.2133 (0.0131)
4	0.25	0.57	0.34			✓	0.2089 (0.0040)
5	1.20	–	1.53		✓		0.1789 (0.0030)

Frequencies in Hz were calculated in three ways: (i) through approximation from chart recordings by experienced clinicians; (ii) through Fourier analysis (the reported frequency corresponds to the peak in the corresponding Fourier spectrum); (iii) from the dominant unstable periodic orbit (UPO). Periodicity categories were based on the attractor reconstructions shown in Fig. 6. Median permutation entropy values are given, with median absolute deviations in brackets.

**Figure 4:**
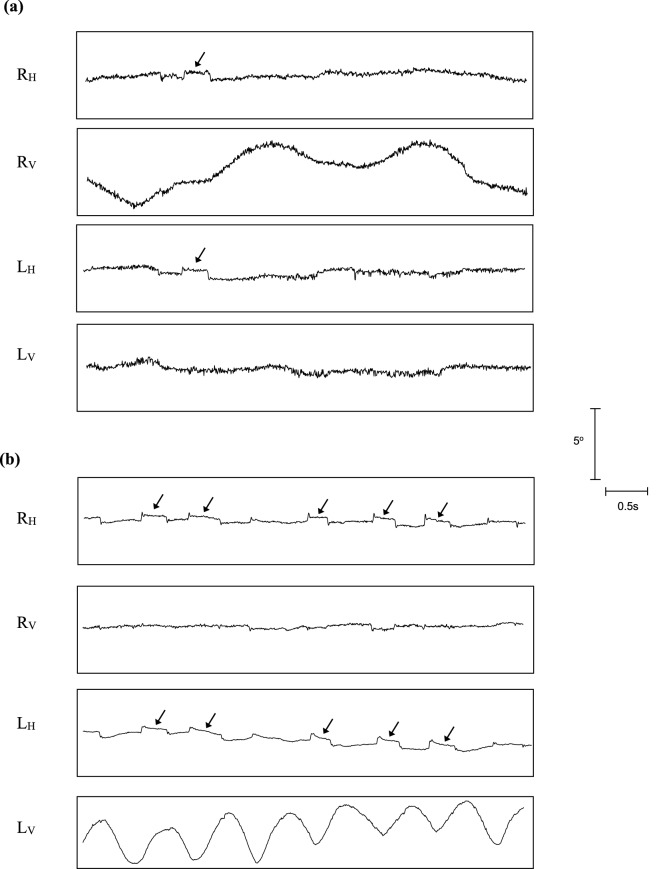
Eye position recordings during binocular viewing of a stationary target located in primary gaze. (**a**) Unilateral vertical nystagmus in the right eye of subject 1. (**b**) Unilateral vertical nystagmus in the left eye of subject 2. R_H_ = right horizontal, R_V_ = right vertical, L_H_ = left horizontal and L_V_ = left vertical. Arrows indicate saccadic intrusions (see text for further details).

**Figure 5:**
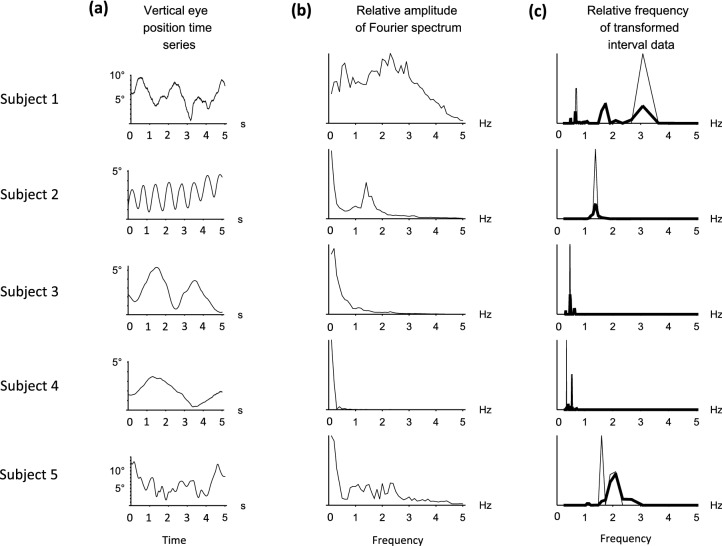
Periodicity analysis. (**a**) 5 s samples of the time series from each subject. See Fig. 3 for the full time courses. (**b**) Frequency spectra. (**c**) Unstable periodic orbits (UPOs). UPOs were determined by comparing the relative frequency of the transformed interval data (thin lines) with surrogate data (bold lines). See text for further details.

Mean amplitudes and frequencies of the uniplanar oscillations in the affected eyes of the 5 subjects ranged from 2.0°–4.0° to 0.25–1.5 Hz, respectively. Subjectively, the time series appeared asymmetrically pendular with downward phases tending to be slower than upward ones*.* Eye position traces for the horizontal and vertical planes for subjects 1 and 2 are illustrated in Fig. 4a,b, respectively. Representative sections of the time series of the affected eyes in the vertical plane for each of the 5 subjects are shown in Figs. 5 (left-hand column (a)) and 6 (left-hand column).

All subjects also exhibited small-amplitude monophasic conjugate horizontal saccadic intrusions in both the oscillating and non-oscillating eyes (see Fig. 4a,b). Amplitudes of the saccadic intrusions ranged between 0.3° and 1.0° and were composed of regular back-to-back saccadic movements. The occurrence and metrics of the monophasic saccadic intrusions in the horizontal plane of the affected eye did not significantly differ from those recorded in the fellow stable eye, or those commonly recorded in the general population^54^.

### The Fourier analysis

The time series and relative amplitudes of the Fourier spectra for each of the 5 subjects are shown in Fig. 5 (first and middle columns, (a) and (b) respectively). Only subject 2 (Fig. 5, second row) displayed a clear periodic behaviour (Table 2). The dominant low frequency components seen in the spectra of the other 4 subjects made it difficult to conclude whether the waveforms were quasiperiodic or nonperiodic solely from the Fourier analysis.

### UPO analysis

The relative frequency of the transformed interval data for all subjects is illustrated in Fig. 5 (right-hand column (c)). The presence of an unstable periodic orbit was determined by comparing the actual function (thin line) with the surrogate function (bold line) of the transformed data. The UPO frequencies computed in this manner are listed in Table 2. Results for each subject will be considered separately:

- Subject 1**—**Two periodic orbits were identified (0.67 Hz and 2.86 Hz), with the lower of the two frequencies showing significance at the 5% level. This lower frequency value was consistent with the findings from the spectral analysis.
- Subject 2—Displayed clear periodic behaviour with an isolated peak in the transformed interval data. A single identifiable periodic orbit was computed at 1.30 Hz (*p* < 0.01).
- Subject 3—Although the time series was very noisy, a periodic orbit peak was identified at 0.47 Hz (*p* < 0.05).
- Subject 4**—**The time series was very noisy, but a periodic orbit peak could be identified at 0.34 Hz (*p* < 0.05).
- Subject 5**—**A clear periodic orbit peak was found at 1.53 Hz (*p* < 0.01).

### Attractor reconstruction and permutation entropy calculations

**Figure 6:**
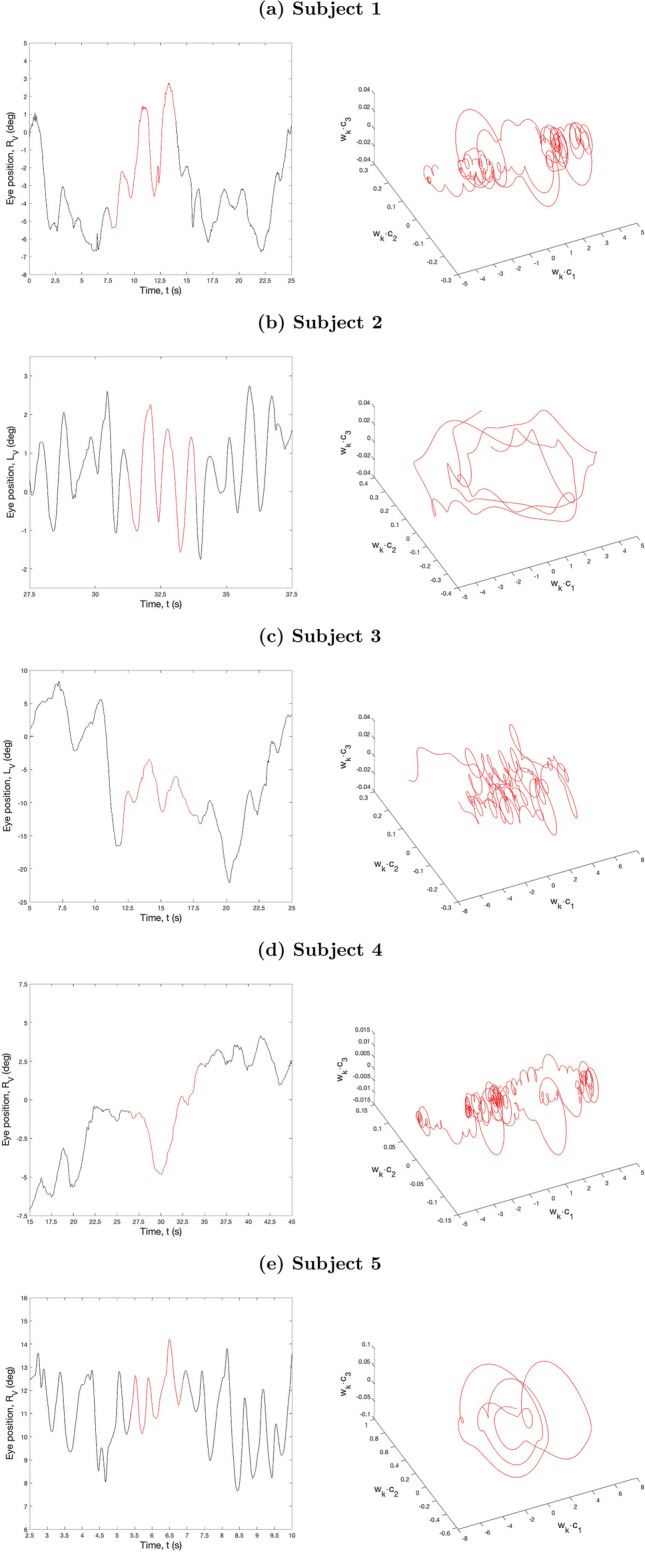
Phase space reconstructions. Left column: Time series segments for each subject showing the unstable periodic orbits (UPO) extracted in each case (red lines). Right column: The attractors reconstructed from the UPOs using delay embedding (see text for further details).

The UPOs and corresponding reconstructed attractors of each time series are shown in the left and right columns of Fig. 6, respectively. It can be observed that subject 2 appears to have a limit cycle attractor, corresponding to a periodic oscillation (Fig. 6b; *cf.* Fig. 2b-top), that subject 5 appears to have a torus attractor, corresponding to a quasiperiodic oscillation (Fig. 6e; *cf.* Fig. 2b-bottom) and that the remaining subjects have more complex, higher-dimensional attractors, corresponding to nonperiodic oscillations (Fig. 6a,c,d; *cf.* Fig. 2c). The classification of subject 2′s attractor as a limit cycle was consistent with both the Fourier and UPO analyses.

**Figure 7:**
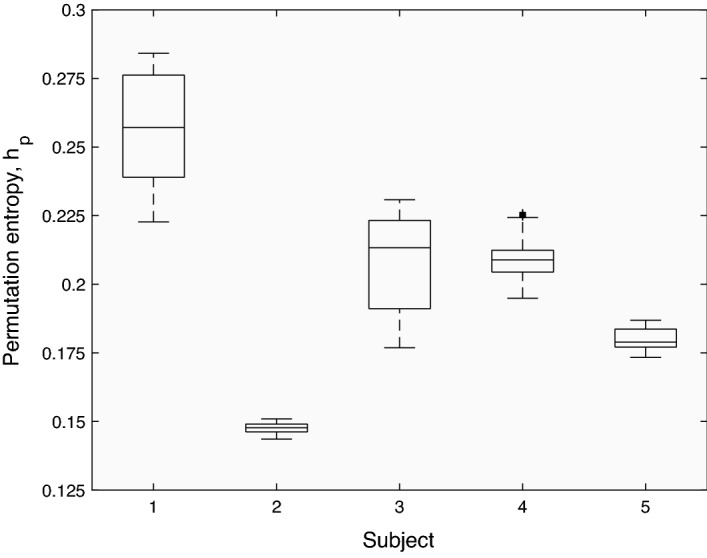
Distributions of permutation entropies obtained by sliding a window across the time series shown in Fig. 3 (see text for further details). In each box plot, the central line denotes the median and the bottom and top edges indicate the 25th and 75th percentiles, respectively. The whiskers show the most extreme data points that are not considered outliers, with outliers plotted as crosses.

The corresponding permutation entropy distributions are shown as box plots in Fig. 7, with medians and median absolute deviations reported in Table 2. The subjects’ median entropy values were statistically distinct (paired Wilcoxon rank sum tests with Bonferroni correction, *p* < 0.01) and gave the following ranking of subjects in terms of increasing waveform complexity (*i.e.* increasing waveform irregularity): 2 → 5 → 4 → 3 → 1. This ranking was consistent with the attractor reconstructions, in that the lowest entropy (subject 2) corresponded to the simplest attractor (limit cycle), the next lowest entropy (subject 5) corresponded to a more complex attractor (torus) and the higher entropies corresponded to attractors with higher dimensionality than the limit cycle and torus. Interestingly, the entropy-based ranking of the subjects’ waveforms with respect to irregularity matched that of an experienced clinician, indicating the potential utility of the measure as a diagnostic index.

## Discussion

### Computational analysis of nystagmus waveforms

This study has presented a computational framework to characterise and quantify ocular oscillations (nystagmus). In this case, the nystagmus was an acquired, late-onset oculomotor instability secondary to a severe monocular visual loss. The monocular vertical ocular oscillations were solely manifest in eyes that had experienced the visual loss. Specifically, we sought to establish the periodicity, attractor type and complexity of the nystagmus time series for each of the subjects. The good agreement between the periodicities found by Fourier analysis and an unstable periodic orbit analysis implies that these periodicities can be determined most simply by readily available spectral analysis software.

Phase space reconstruction allowed attractor identification and the computation of a time series complexity measure, the permutation entropy. The permutation entropy index was able to differentiate between a periodic oscillation associated with a limit cycle attractor, a quasiperiodic oscillation associated with a torus attractor and nonperiodic oscillations associated with higher-dimensional attractors. Importantly, the permutation entropy was able to rank the oscillations in order of complexity, thereby providing an objective index of nystagmus oscillation complexity that could not be obtained via UPO analysis or attractor identification alone.

### Classifying nystagmus waveforms

Many attempts have been made to classify nystagmus time series. In the case of an early onset (infantile) nystagmus, waveforms have been closely associated with their fast and slow phases^8, 9, 11, 17, 20^. More recently the application of principal component analysis revealed that 97% of the variance of the waveforms can be described by a linear sum of 2 component waveforms (*i.e.* sawtooth and pseudocycloid)^35^. In addition, other groups have proposed that a waveform classification should also take account of the metrics of the low-velocity foveation periods that are often a constituent of the waveforms^5, 7–9, 11–14, 55^.

In the case of a non-HBP late-onset (acquired) nystagmus, the task is somewhat more complex due to the underlying pathologies. Waveforms show marked spatial and temporal variation and, on occasion, display behaviours pathognomonic of the site of the neural disorder^4^. Notwithstanding, we suggest that our computational framework will provide the analytical basis to classify both early- and late-onset nystagmus waveforms.

It is important to note that in parallel with previous investigations of nystagmus waveforms, there have been numerous studies exploring the perceptual consequences of early-onset and late-onset nystagmus. In nystagmus, the moving retinal image reduces many aspects of visual performance^4, 6, 7, 56^. Specifically, both early- and late-onset subjects not only experience losses in visual acuity, but the latter group also experience the disturbing movement of their visual environment (oscillopsia). It is these visual consequences which have driven us to understand the underlying mechanisms of nystagmus to seek specific dedicated therapeutic management (see “Concluding remarks” section).

### Mechanisms underlying the waveform complexity

In previous studies we investigated whether early-onset (congenital/infantile) nystagmus is generated by a deterministic mechanism^26–28^, finding evidence for periodic behaviour contaminated by noise^24^. This finding, supported by a Fourier analysis, indicated a single distinct peak in the UPO spectrum of each subject^16, 24^. Although nonlinear deterministic systems that do not settle into steady state behaviour, can show periodic, quasiperiodic or chaotic behaviour, this would be associated with a skeleton of one or more commensurate periodic orbits, at least two non-commensurate periodic orbits or a multiplicity of periodic orbits respectively. In our present cohort of a late-onset nystagmus, we commonly found cases showing multiple unstable periodic orbits, although only one orbit was statistically significant in each case. This suggests that the data noise prevented the UPO analysis from directly distinguishing between these different oscillation categories, requiring the attractor reconstruction to do so.

### Concluding remarks

In the past, the standard clinical assessment of the basic metrics of a nystagmus has proved valuable, but somewhat limited^57^. Here we show how the application of a nonlinear dynamical systems approach can substantially improve our understanding of the characteristics of the nystagmus (irrespective of its onset-time). Specifically, permutation entropy analysis has been demonstrated as a potentially valuable tool for monitoring a nystagmus system’s dynamics over time, with scope for assisting differential diagnosis. As such, application of the index to the infantile nystagmus waveforms we have considered in our previous studies would be a natural extension of the work presented here. However, a decision-support system of this type would require the analysis of a much larger cohort of nystagmus subjects, in order for classification boundaries between different oscillation types (and their mapping to visual loss severity) to be robustly derived. In this regard, the permutation entropy index could prove particularly useful in assessing the maturation and adaptation of nystagmus waveforms in early and late onset nystagmus, the quantifying of changes following therapeutic management (*e.g.* drug and gene therapy^58, 59^, extraocular surgery^17, 25, 59^, biofeedback^60^, external periodic forcing^61^), whilst also providing an additional tool for future oculomotor systems modelling.

It is also important to stress that any investigation of a nystagmus time series is greatly dependent on the manner of its recording, storage, analysis and interpretation. Presently, these tasks are more easily achievable in a laboratory environment. We are therefore greatly encouraged by the improved availability of clinic-friendly 2- and 3-dimensional eye movement recording systems^62, 63^. Their high resolution, greater linearity, improved sampling rates and noise reduction properties are particularly important for examining nystagmus waveforms, which can vary from cycle to cycle. Advances in signal calibration of a moving eye^64, 65^, together with new techniques for noise reduction of the data^66^, will no doubt assist in the analysis of a nystagmus time series. Moreover, targeted studies on nystagmus feature extraction^9, 20, 35, 67^ and modelling of nystagmus waveforms (see “Mechanisms underlying the waveform complexity” section) will improve our understanding of the mechanisms underpinning the oscillations. We believe that, with nonlinear time series analysis methods becoming more established^68^ and the integration of automated techniques for data mining and decision support (machine learning)^69, 70^, the clinical assessment, management and modelling of nystagmus is entering a new and rewarding phase.
